# Physiological Measures of Dopaminergic and Noradrenergic Activity During Attentional Set Shifting and Reversal

**DOI:** 10.3389/fpsyg.2018.00506

**Published:** 2018-04-11

**Authors:** Péter Pajkossy, Ágnes Szőllősi, Gyula Demeter, Mihály Racsmány

**Affiliations:** ^1^Institute of Cognitive Neuroscience and Psychology, Research Centre for Natural Sciences, Hungarian Academy of Sciences, Budapest, Hungary; ^2^Department of Cognitive Science, Budapest University of Technology and Economics, Budapest, Hungary; ^3^Rehabilitation Department of Brain Injuries, National Institute of Medical Rehabilitation, Budapest, Hungary

**Keywords:** pupil size, eye-blink rate, attentional set shifting, executive functions, dopamine, noradrenaline

## Abstract

Dopamine (DA) and noradrenaline (NA) are important neurotransmitters, which are suggested to play a vital role in modulating the neural circuitry involved in the executive control of cognition. One way to investigate the functions of these neurotransmitter systems is to assess physiological indices of DA and NA transmission. Here we examined how variations of spontaneous eye-blink rate and pupil size, as indirect measures of DA and NA activity, respectively, are related to performance in a hallmark aspect of executive control: attentional set shifting. We used the Intra/Extradimensional Set Shifting Task, where participants have to choose between different compound stimuli while the stimulus-reward contingencies change periodically. During such rule shifts, participants have to refresh their attentional set while they reassess which stimulus-features are relevant. We found that both eye-blink rate (EBR) and pupil size increased after rule shifts, when explorative processes are required to establish stimulus–reward contingencies. Furthermore, baseline pupil size was related to performance during the most difficult, extradimensional set shifting stage, whereas baseline EBR was associated with task performance prior to this stage. Our results support a range of neurobiological models suggesting that the activity of DA and NA neurotransmitter systems determines individual differences in executive functions (EF), possibly by regulating neurotransmission in prefrontal circuits. We also suggest that assessing specific, easily accessible indirect physiological markers, such as pupil size and blink rate, contributes to the comprehension of the relationship between neurotransmitter systems and EF.

## Introduction

The adaptive control of behavior and information processing encompasses the flexible shift of attentional focus, updating of relevant information and the inhibition of irrelevant information (Miyake et al., 2000). This broad set of control functions, often referred to as executive functions (EF), evolves from the interaction of complex brain networks (Robbins and Rogers, 2000). A wealth of research suggests that areas in the prefrontal cortex and their interactions with subcortical networks play the most vital role in implementing executive control functions (Alexander et al., 1986; Smith and Jonides, 1999; Frank et al., 2001; Miller and Cohen, 2001; Chudasama and Robbins, 2006). Importantly, the modulatory effect of subcortical areas on the prefrontal cortex is thought to be implemented through neurotransmitter systems which influence neural processing in several areas of the prefrontal cortex (Doya, 2008; Robbins and Arnsten, 2009). In this paper, we aimed to focus on two neurotransmitters, dopamine (DA) and noradrenaline (NA), to show how within- and between-subject variability of DA and NA levels, measured by physiological markers, is related to attentional set shifting, a specific component of EF.

Attentional set can be defined as a class or dimension of environmental features, which is considered as being task-relevant and is attended by the individual (Owen et al., 1993; Heisler et al., 2015). The ability to switch between attentional sets, as a response to current task demands, is an important aspect of cognitive flexibility. This executive function is often measured by the Wisconsin Card Sorting Task (henceforth WCST; Berg, 1948; Heaton et al., 1993), and by the Intra/Extradimensional Set Shifting Task (henceforth IEDT; Downes et al., 1989; Owen et al., 1992). It is widely shown that successful performance in these tasks is associated with activations in the prefrontal cortex (see e.g., Milner, 1963; Janowsky et al., 1989; Grafman et al., 1990; Dias et al., 1996; Hampshire and Owen, 2006; Nyhus and Barceló, 2009). Furthermore, a substantial body of neuropsychological evidence revealed that attentional set shifting is impaired in several psychiatric and neurological conditions (schizophrenia: Heinrichs and Zakzanis, 1998; Jazbec et al., 2007; Reichenberg and Harvey, 2007; Pantelis et al., 2009; obsessive–compulsive disorder: Roh et al., 2005; Chamberlain et al., 2006; Demeter et al., 2013; Parkinson’s disease: Owen et al., 1992; Kudlicka et al., 2011).

In both the WCST and the IEDT, participants have to choose between complex stimuli characterized by distinct stimulus dimensions (e.g., in the IEDT, figures with different shapes overlaid by lines with different curvature). Only one feature of one stimulus-dimension is rewarded (e.g., rectangle shape), and the task is to find out the rewarded feature through trial and error learning. In doing so, participants have to specify which stimulus dimension they attend to, thereby creating an attentional set. Importantly, after participants have managed to figure out the stimulus–reward contingency, as indicated by consecutive correct choices, the reward rule changes: the rewarded stimulus becomes either another feature from the same dimension (e.g., a different figure), or a feature from another dimension (e.g., one of the lines). In the former case, reversal learning is required: participants have to ignore the previously rewarded stimulus and turn to a previously non-rewarded one. In the latter case, participants have to reassess which dimension they attend to – this process is termed attentional set shifting.

Two conceptually distinctive phases can be identified in both the IEDT and the WCST. First, participants have to figure out the rewarded feature through trial and error learning based on feedback received for previous choices. After the rule changes, they have to explore what the new rewarded feature is. This can be termed the *explorative phase* of the task. Second, after the stimulus–reward contingencies are identified, participants have to continuously choose the correct response option based on the established attentional set. That is, participants have to exploit the acquired knowledge to choose the correct option. This can be labeled as the *exploitative phase* of the task.

Importantly, both NA and DA transmissions are linked to exploration and exploitation. NA is released throughout the cortex, and this NA transmission originates almost exclusively from the brain stem nucleus locus coeruleus – often termed the LC/NA system (Aston-Jones et al., 1999; Aston-Jones and Cohen, 2005). The adaptive gain theory, proposed by Aston-Jones and Cohen (2005), differentiates between a phasic and a tonic mode of LC function. The phasic mode is associated with a moderate tonic firing level and with strong task-related phasic burst of LC, which serves to coordinate cortical networks in order to facilitate task-relevant responses. In contrast, the tonic mode, associated with exploration and task-disengagement, is characterized by a high tonic firing level and by the absence of clear phasic bursts. Partly similar theories also suggest that phasic NA transmission is related to the coordination of task relevant networks (Bouret and Sara, 2005), whereas tonic NA firing represents the level of unexpected uncertainty (Yu and Dayan, 2005), which in turn leads to explorative behavioral tendencies.

The functions attributed to DA transmission are also relevant for the regulation of explorative and exploitative behavioral tendencies. Specifically, reinforcement learning required for establishing the correct stimulus–reward contingency is suggested to be dependent on midbrain DA neurons. These neurons are thought to code reward prediction error, that is, the difference between the expected and the experienced reward (Schultz et al., 1997; Glimcher, 2011). In current theoretical models, these low-level features of individual DA neurons underlie the regulatory function of the DA neurotransmitter system. Through different paths involving different DA receptors, DA might regulate the balance between stability and flexibility of cortical representations (Frank and O’Reilly, 2006; Cools and D’Esposito, 2011; Maia and Frank, 2011). Furthermore, it is also suggested that tonic changes in striatal DA outflow might contribute to the regulation of the trade-off between exploration and exploitation (Frank et al., 2009; Beeler et al., 2010; Humphries et al., 2012). Interestingly, in some computational models, it was proposed that the striatal DA system, which is involved in reinforcement learning, might interact with the LC/NA system to determine the shift between exploitation and exploration (McClure et al., 2006; Frank et al., 2007).

Based on the above, both NA and DA transmission can contribute to attentional set shifting through the above described explorative or exploitative processes, respectively. Accordingly, supporting evidence for a link between attentional set shifting and NA/DA transmission has already been reported. In rodent studies, the manipulation of both NA and DA transmission affects attentional set shifting (Lapiz and Morilak, 2006; Tait et al., 2007; McGaughy et al., 2008; Cybulska-Klosowicz et al., 2017a,b). Moreover, in human studies using the WCST, individual differences in task performance are associated with neurobiological substrates linked to DA transmission (e.g., Joober et al., 2002; Hsieh et al., 2010). These studies either manipulate the level of DA and NA before the task or observe the consequences of individual differences. In our study, we aimed to enrich the above evidence by using a method which enables us to track online changes in neurotransmitter level. Therefore, we assessed easily accessible physiological indices which indirectly measure the activity of both the NA and the DA system (for a similar approach, see Van Slooten et al., 2017). Investigating the link between such measures and task performance might shed light on factors which determine individual differences in attentional set shifting.

NA transmission was investigated by assessing pupil diameter, as recent results suggest that pupil size reflects the activity of the LC (Aston-Jones and Cohen, 2005; Murphy et al., 2014; Joshi et al., 2016). Sudden, task-evoked increase in the size of the pupil has been the subject of scientific inquiry for decades, in particular as a measure of mental effort or cognitive load (Kahneman and Beatty, 1966; Beatty, 1982). Such pupil dilation accompanies various types of cognitive processing and is associated with phasic bursts of the LC/NA system (Aston-Jones and Cohen, 2005; Gilzenrat et al., 2010). Recently, more enduring, slower pupil size changes during cognitive processing have also attracted attention (Jepma and Nieuwenhuis, 2011; Hayes and Petrov, 2015). Interestingly, pupil size might signal a third aspect of NA function: as it was revealed by a current study (Tsukahara et al., 2016), individual variations in baseline pupil size, measured before the task begins, were associated with working memory performance. They found that the pupil size of participants with high working memory capacity was larger, as compared to participants with low working memory capacity. The authors suggest that such task-unrelated baseline measure of pupil size might be an index of large-scale brain network activity orchestrated by the LC/NA system.

As an indirect index of DA transmission, we assessed eye-blink frequency. Spontaneous eye-blink rate (EBR) is affected by DA agonist and antagonists (e.g., Blin et al., 1990; Cavanagh et al., 2014), and disorders characterized by atypical DA levels are associated with differences in EBR (decrease in Parkinson’s disease: e.g., Karson et al., 1984; Bologna et al., 2012; increase in schizophrenia: e.g., Helms and Godwin, 1985; Swarztrauber and Fujikawa, 1998). Although the underlying mechanisms and characteristics of this link are still unclear, and the status of EBR as a biological marker of DA is disputed (e.g., van der Post et al., 2004; Tharp and Pickering, 2011), several lines of evidence point out that EBR might be a useful indirect index of striatal DA transmission (for reviews, see Jongkees and Colzato, 2016; Eckstein et al., 2017). It has been suggested that higher EBR might indicate lower updating threshold for cortical representations, which then leads to flexibility in processing, but at the cost of distractibility (Jongkees and Colzato, 2016).

This baseline EBR^1^ is usually measured under free viewing condition with no specific task instruction. When EBR is measured under a specific task, both increase and decrease of EBR can be observed, as compared to rest periods. Before and after eye-blinks, visual processing is suppressed (Manning et al., 1983; Stevenson et al., 1986), and tasks involving visual attention typically decrease EBR (Drew, 1951; Stern and Skelly, 1984). Furthermore, several tasks involving mental effort are associated with within-task EBR changes (Holland and Tarlow, 1975; Bentivoglio et al., 1997; De Jong and Merckelbach, 1990; Siegle et al., 2008; Oh et al., 2012). Although most of the above studies did not link EBR changes to DA, some recent studies showed that task-related changes in EBR might specifically signal changes in DA transmission (van Bochove et al., 2013; Peckham and Johnson, 2016; Rac-Lubashevsky et al., 2017). This points to the possibility that EBR is not only a baseline measure of DA transmission, but it is suitable for tracking within-task changes of DA level.

Altogether several lines of evidence indicate that pupillometry and the measurement of EBR are possible indirect indices of NA and DA levels, and are suitable measures for revealing how these neurotransmitters are involved in explorative and exploitative aspects of attentional set shifting performance. In a recent study (Pajkossy et al., 2017), we have demonstrated that there is a relationship between tonic pupil size and attentional set shifting. Participants performed eye-tracker adapted versions of both the IEDT and the WCST. We found that pretrial pupil size increased in the explorative phase of the tasks, whereas in the exploitative phase of the tasks, a steady decrease in pretrial pupil size was observed. In the present study, we aimed to replicate these findings. Furthermore, we predicted that baseline pupil size, measured in a similar way as in Tsukahara et al. (2016), would be related to task performance. In line with their findings, we predicted a positive correlation between baseline pupil size and task performance.

Regarding EBR and DA transmission, we aimed to test whether we could use EBR as an indirect measure of DA transmission during information processing (and not under passive viewing conditions). First, we tested whether average EBR during the task (i.e., baseline EBR) could be linked to task variables. Second, we examined whether EBR changes did accompany rule shifts. As EBR is suggested to index the balance between stability and the flexibility of cortical representations, our predictions were similar to that of pupil size changes: a steady decrease during the exploitative phase, when the positive feedback acted to maintain current representations, whereas a sudden increase during the explorative phase, when the flexible updating of cortical representations was required.

## Materials and Methods

### Participants

Participants were Hungarian undergraduate students, who received a monetary reward for their participation. We asked participants to refrain from consumption alcohol, caffeine, and nicotine one day prior to the experiment, because these substances might affect physiological variables, like EBR and pupil size (Holmqvist et al., 2011). Participants who did not comply with this instruction were not included in the study. The initial sample size was 60 participants. Two participants were excluded due to diagnosed neurological conditions, whereas a further five participants were excluded due to recording errors resulting in substantial loss of eye-tracking data. Furthermore, to ensure reliable blink detection, we took a rather conservative approach and excluded five participants due to low eye-tracker data quality. Thus, the final sample size consisted of 48 participants (29 females; age range: 18–31 years, *M*_age_ = 22.0, *SD* = 2.4). All experiments were run between 10 a.m. and 5 p.m., as EBR is affected by the circadian rhythm with increased EBR in the evening (Barbato et al., 2000; Jongkees and Colzato, 2016).

### The IEDT Task

#### Structure of the Task

**FIGURE 1:**
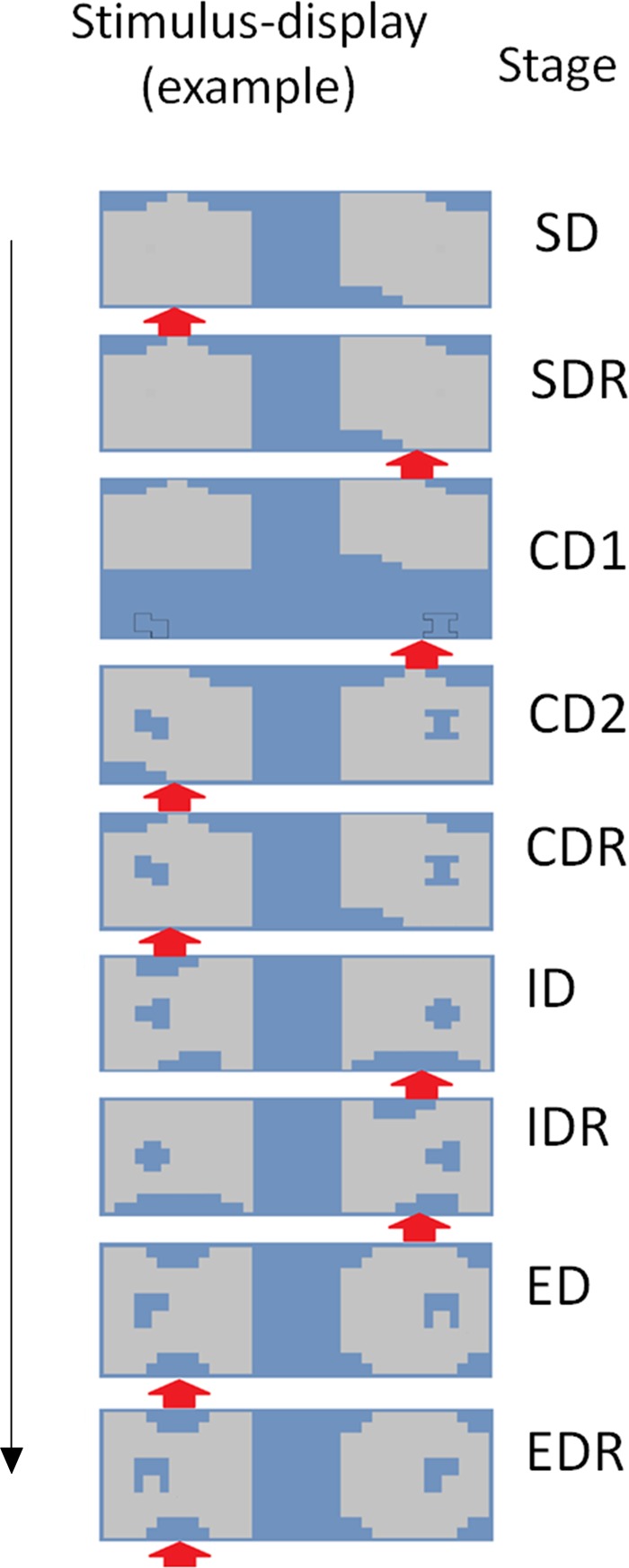
The eye-tracker adapted version of the Intra/Extradimensional Set Shifting Task. One possible trial from each stage is presented. The exemplars of the two stimulus-dimensions are randomly paired with each other in each trial (see, e.g., the pairings of large and small figures in the ED and the EDR stages). Red arrow shows which compound stimuli were rewarded in the specific trial. The stimulus exemplars change after the CDR and the IDR stage. During the first seven stages, one of the large shapes is rewarded, whereas in the last two stages, one of the small shapes is rewarded. SD, Simple Discrimination; SDR, Simple Discrimination Reversal; CD1, Compound Discrimination 1; CD2, Compound Discrimination 2; CDR, Compound Discrimination Reversal; ID, Intradimensional Set Shifting; IDR, Intradimensional Set Shifting Reversal; ED, Extradimensional Set Shifting; EDR, Extradimensional Set Shifting Reversal.

The most frequently used IEDT version is part of the Cambridge Automated Test Battery (henceforth CANTAB), an often used neuropsychological test battery (Fray et al., 1996). In this task, stimulus dimensions are spatially overlapping. We adapted this task to eye-tracking by spatially segregating the two stimulus dimensions (holes inside figures, see **Figure 1**), which enabled us to independently track attention regarding the two stimulus dimensions. In all other aspects, the task was identical to the IEDT used in the CANTAB. Importantly, to equate net luminance of the screen, all figures and holes had the same surface size.

In each trial, participants had to choose the correct stimulus from two compound stimuli with two stimulus dimensions – two large rectangular figures with holes inside (in the following, we will refer to the rectangular figures as *large* figures, whereas the holes inside will be labeled as *small* figures). Participants were instructed to use the feedback received for previous choices to figure out the reward–stimulus contingency. We also told them that after consecutive correct responses, the rule would be changed. This rule shift was not signaled to them, but could be figured out based on the feedback received (i.e., using the previously correct stimulus–reward contingency led to negative feedback after rule shift).

The task consisted of nine stages. In some of the stages, only the stimulus–reward contingency changed, whereas in other stages, new stimuli were also introduced. The same stimulus-exemplars were shown in all trials of a specific stage (i.e., the same two large and small figures), but their pairing varied randomly (i.e., both small figures could be presented on the surface of both large figures). The only constraint was that the same pairing could be presented only five times consecutively. In each phase, only one stimulus-exemplar was rewarded (e.g., one large figure). Participants advanced through the stages by figuring out the correct stimulus–reward contingency, and by choosing always the correct stimuli (i.e., the compound stimuli which included the rewarded stimulus-exemplar). Six consecutive correct responses triggered a rule shift and the start of the next stage. If this criterion was not achieved after 50 trials, then the task was terminated.

During the nine stages, the stimuli and the rule shifts were varied systematically to test different aspects of cognitive processing (see **Figure 1**). In the first two stages, only the large shapes were presented. One of the large figures was randomly selected to be rewarded in the first stage (simple discrimination, SD), and the other large figure became rewarded in the second stage (simple discrimination reversal, SDR). This large figure remained the rewarded stimulus-exemplar during the next two stages (compound discrimination 1–2, CD1–CD2), where the two small figures were introduced gradually: in the third stage, the two stimulus-dimensions were presented in distinct areas of the screen, whereas in the fourth stage, they formed a compound stimulus – this arrangement was used in the later stages of the task. In the fifth stage, the large figure rewarded previously in the SD stage became the rewarded stimulus-exemplar again (compound discrimination reversal, CDR). Thus, the SDR and CDR stages constituted an example of *reversal learning*: a previously non-rewarded exemplar of a stimulus dimension became rewarded.

The more complex part of the task started in the sixth and seventh stage, where new large and small figures were introduced. In the sixth stage, one of the new large figures was randomly chosen to be rewarded (intradimensional set shifting, ID), whereas in stage 7, the other large figure was rewarded (ID reversal, IDR). Then, in the final two stages, a third set of large and small figures was introduced. Importantly, however, this time the small figures became the rewarded features – one of the new small figures was chosen to be rewarded in the eighth stage (extradimensional set shifting, ED), whereas the other small figure in the ninth stage (ED reversal, EDR). This part of the task tested three different cognitive functions. First, in the IDR and EDR stages, again reversal learning was required. Second, in the ID stage, *intradimensional set shifting* was required: attention had to be directed to new exemplars of the previously attended stimulus-dimension. Third, the ED stage required *extradimensional set shifting*: attention had to be transferred to the exemplars of a previously unattended stimulus dimensions.

#### Structure of a Trial

Each trial started with a 2.5-s *fixation cross period*, during which participants had to fixate a yellow fixation cross on a blue background. This was followed by the *stimulus presentation period*, when the two compound stimuli were presented on the left and the right side of the screen. The participants indicated their choice by clicking either the left or the right mouse button. After the response, a 0.5-s blank screen with blue background followed, and then feedback was given for 1 s. During the *feedback period*, the screen layout of the presentation period was shown again, but this time, a green or a red frame appeared around the compound stimuli, indicating correct or incorrect choice, respectively. Different sound signals were also associated with both the correct and the incorrect choices.

### Eye-Tracking

We used an SMI RED500 remote eye-tracker, with a sampling rate of 250 Hz. No chin rest was used, and data from both eyes were recorded.

#### Eye-Blink Data Preprocessing

Blink data were also derived using the eye-tracker data. We used the algorithm of the SMI Begaze data processing software (Sensomotoric Instruments, Teltow, Germany) to detect eye-blinks, and the identified eye-blinks were then further processed using MATLAB (MathWorks, Inc., Natick, MA, United States). At the initiation of an eye-blink, the eye-lid occludes an increasing area of the pupil. Due to specifics of saccade and fixation detection, the algorithm detects a downward saccade at this time point. At the end of an eye-blink, the eye-lid is gradually lifted, and the pupil can be detected – resulting in the detection of an upward saccade. Eye-blinks are then detected as periods without detectable pupil surrounded by a downward and an upward saccade.

This detection method is a more indirect way to detect eye-blinks than electrooculography or video-based methods, and is sensitive to eye-tracking data quality. Thus, data were carefully preprocessed and we took a conservative approach to avoid false detection of eye-blinks. First, we examined noise levels in the gaze data point used by the detection algorithm. During fixations, the gaze direction remains relatively stable, and thus variations in reported gaze point might be attributable to a large extent to measurement noise. Thus, we calculated the root mean square error (RMS error), a measure of variation for each fixation and for each participant (Holmqvist et al., 2011). The median of RMS error values for each participant was then computed. The sample mean of these median values was *M* = 0.1, *SD* = 0.1. This falls within the range of noise levels reported for remote eye-trackers (Holmqvist et al., 2011; Orquin and Holmqvist, 2017). Nevertheless, we excluded five participants, whose median RMS error value exceeded the sample mean by two SDs. We used this rather strict criterion to ensure correct detection of eye-blinks. As a second measure ensuring reliable detection, we only accepted data points as eye-blinks, where data from both eyes indicated the presence of an eye-blink.

Examination of individual eye-blink duration distributions revealed that only a few eye-blinks lasted less than 60 ms (0.5% of all blinks). These eye-blinks were labeled as measurement artifacts, and were discarded from further analysis. Following previous research (von Mühlenen et al., 2013), eye-blinks above 500 ms were also discarded (2.8% of all blinks).

We computed descriptive statistics of eye-blink data generated using the above preprocessing steps for the final sample. The sample mean for median eye-blink duration was 114.6 (*SD* = 21.0), with an interquartile range 96 to 150 (computed for the final sample, *N* = 47). The mean eye-blink frequency during the task was 12.3 (*SD* = 9.3). The validity of our measurement is supported by the fact that these values are similar to previous findings for both eye-blink duration (Jandziol et al., 2001; von Mühlenen et al., 2013) and EBR (for a review, see Jongkees and Colzato, 2016).

#### Pupil Data Preprocessing

Noise in pupil data was also filtered out. During eye-blinks, the eye-lid occludes parts of the pupil and alters pupil size. Thus, we identified eye-blinks in our data, as described in the previous section, and removed pupil data during eye-blinks. High-magnitude changes in pupil size before the start and after the end of eye-blinks were also removed from the data. Finally, segments of missing data points were also removed. These steps resulted in the removal of an average 6.2% (*SD* = 4.9) of our data. Missing data points were replaced using linear interpolation. Thereafter, to filter high frequency noise, we deleted those pupil size values in each data set, which deviated from the mean of the data set by more than three SDs. Ten data points before and after such segments were also removed. On average, less than 1% of the data were removed this way. These missing data points were also replaced by linear interpolation. Finally, data were smoothed using a Savitzky–Golay filter (parameters: polynomial order: 2, frame size: 21).

### Statistical Analysis

The link between task performance and physiological measures was assessed from two different aspects. First, we investigated how individual differences in baseline values of pupil size and EBR are associated with task performance. Second, we also examined how EBR and pupil size change before and after rule shifts (i.e., during the exploitative and the explorative phase of the task). In both cases, an important aspect of the analyses considered the ED stage; thus, six participants who failed to reach the ED stage, were excluded from analysis. Furthermore, the first stage was considered as a warm-up phase, thus data from this stage were not involved in any of these analyses.

#### Analysis of Individual Differences

We analyzed two important aspects of task performance. First, we computed the number of errors made between the SDR and the ID stages. This measure indexes the ability to learn from feedback in a task involving a set of changing complex stimuli, but involves no ED. In contrast, we also computed the errors made during the eighth stage, where ED is required. This measure specifically indexes the ability to disregard the attentional set, which was relevant for the previous seven stages, and flexibly adapt a new attentional set.

To assess between-subjects variation in pupil size, before the start of the IEDT, we asked participants to fixate a fixation cross at the center of the screen for four seconds, and then we computed average pupil size during this period (similar assessment was used in Tsukahara et al., 2016). To measure individual differences in EBR, we calculated blinking rate during the task: we divided the number of eye-blinks starting from the SDR stage until the last stage with the time (in minutes).

The behavioral measures of task performance were not normally distributed due to the ceiling effect. Thus, we computed Spearman rank correlation to investigate the relationship between behavioral performance and physiological indices.

#### Analysis of Rule Shifts

To investigate changes during the task, we first computed EBR and pupil size values for each trial period separately (stimulus presentation, feedback, and fixation cross). EBRs were computed for each trial period by dividing the number of eye-blinks during the period by the trial period time in seconds (trial time was fixed for the feedback and fixation cross period, but varied during the stimulus presentation period). Note that this computation differs from the computation of baseline EBR, where the scale of time data was in minutes. Pupil size values for each trial period were computed by calculating the mean pupil size of that period.

**FIGURE 2:**
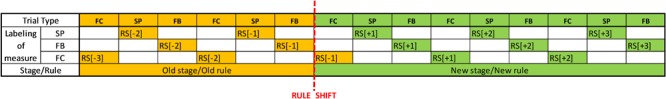
Labeling of measurement points for analysis of different trial periods. FC, Fixation cross period; SP, Stimulus presentation period; FB, Feedback period; RS[–1], RS[–2], RS[–3], the first, second, or third trial, respectively, preceding the rule shift; RS[+1], RS[+2], RS[+3], the first, second, or third trial, respectively, following the rule shift.

To analyze the transition between the exploitative and explorative phases, we focus predominantly on the fixation cross period. During stimulus presentation and feedback, EBR and pupil size might be influenced both by neurotransmitter levels and by visual features of the presented stimuli. During the fixation cross period, however, visual changes are absent; thus changes in these measures might be attributable to changes in neurotransmitter levels. Nevertheless, as fixation cross periods are embedded into a stimulus presentation–feedback–fixation cross cycle, the other two periods are also investigated. For all three periods, measurement points are categorized according to their position relative to a rule shift. We use a corresponding labeling throughout the article. For example, the first trial after a rule shift will be labeled using the denotation RS[+1] (RS standing for rule shift), whereas we will refer to the third trial preceding a rule shift using the denotation RS[–3]. The assignment of these labels around the rule shift is depicted in **Figure 2**. Note that the fixation cross period directly preceding the first stimulus presentation of a new stage counts as RS[–1], as participants have no information at that time point that the rule has changed.

Values of pupil size and EBR are averaged across all rule shifts for different trial-types (e.g., RS[–1] or RS[+2]). For example, we averaged the values of pupil size for all RS[–1] trials the different rule shifts, get an average RS[–1] value. Because the first stage was constituted as a warm-up phase, data from the first rule shift (between SD and SDR states) were not involved in this calculation. Reversal and attentional set shifting constitute the two fundamental shift types; thus these average values were computed separately for the reversal stages (transition to the CDR, the IDR, and the EDR stage), and for the stages where attentional set shifting is required (transition to the ID and the ED stage). Note that during rule shifts requiring reversal, rule shifts can be detected based on negative feedback for responses based on the outdated stimulus–response contingency. In contrast, during rule shifts requiring attentional set shifting, rule shift is signalized by the stimulus-layout change, and no feedback processing is required.

For the exploitative phase (i.e., before rule shift), we predicted for both EBR and pupil size a steady decrease. In the case of pupil size, this was analyzed using a repeated measures ANOVA with trial as an independent factor (RS[–6] to RS[–1]). The skewed distribution of EBR did not allow the use of ANOVA, thus we used its nonparametric variant, the Friedman ANOVA. At the beginning of the explorative phase, we predicted a sudden increase in both pupil size and EBR. In the case of pupil size, we compared RS[–1]and RS[+1] using a paired sample *t*-test, whereas for EBR, due to violation of the normality assumption, the Wilcoxon signed rank test was used.

#### Confounding Factors

Both pupil size and EBR are physiological measures, which might be influenced by several factors (e.g., arousal level, health conditions, motivational factors). During debriefing, we assessed these factors: we asked participants about sleep hours and sleep quality. They also completed the Karolinska Sleepiness Scale (Åkerstedt and Gillberg, 1990) to assess their drowsiness and fatigue. Moreover, participants rated on a nine-point scale how much effort they exerted during the IEDT. We examined whether these factors are related to EBR or pupil size to reveal whether these factors should be considered as confounders. Five participants indicated to take some form of medication (e.g., contraceptives or antibiotics), whereas 10 participants indicated to suffer from a minor cold. These factors were not considered as exclusion criteria, thus these participants were not excluded from our sample. Nevertheless, to rule out that our results are not confounded by these factors, all the analyses described above were also rerun without these participants, to check whether the pattern of results changes when these subjects are excluded.

## Results

### Behavioral Results

**Table 1** presents mean number of trials required to pass each stage and the number of participants passing the stages. As expected, the most difficult part of the task was the ED stage. Here, on average, 21.3 trials (*SD* = 15.3) were required to pass the stage. In contrast, in other stages, the trials to criterion mean values varied in around 10 trials.

**Table 1:** Performance in the intra/extradimensional set shifting task

Measures	SD	SDR	CD1	CD2	CDR	ID	IDR	ED	EDR
Trials to criterion	8.35	9.37	10.87	8.73	9.63	9.67	10.49	21.26	8.22
	(7.09)	(6.97)	(11.38)	(7.18)	(4.36)	(8.76)	(9.20)	(15.28)	(2.39)
Pass-Nr	48	48	47	44	43	43	43	42	36
Wilcoxon-Z (comparing with last stage)	–	3.04^∗∗^	0.42	0.10	3.05^∗∗^	1.84^+^	1.52	4.36^∗∗∗^	4.41^∗∗∗^

*Trials to criterion: number of trials/choices required to complete a stage (values represent the mean values, standard deviations are shown in parentheses); Pass-Nr, number of participants successfully completing a stage; SD, Simple Discrimination; SDR, Simple Discrimination Reversal; CD1, Compound Discrimination 1; CD2, Compound Discrimination 2; CDR, Compound Discrimination Reversal; ID, Intradimensional Set Shifting; IDR, Intradimensional Set Shifting Reversal; ED, Extradimensional Set Shifting; EDR, Extradimensional Set Shifting Reversal; ^∗∗^*p* < 0.01; ^∗∗∗^*p* < 0.001, ^+^*p* < 0.10.*

### Analysis of Individual Differences

**FIGURE 3:**
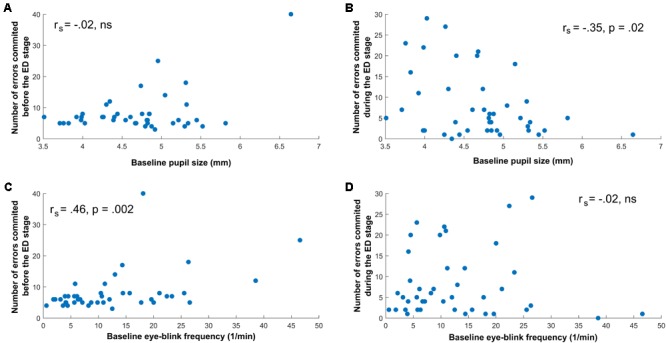
Relationship between Task-Performance Measures, Pupil size, and Eye-Blink Rate. **(A)** No significant correlation between baseline pupil size and errors before the ED stage. **(B)** Significant negative correlation between baseline pupil size and errors during the ED stage. **(C)** Significant positive correlation between baseline eye-blink rate and errors before the ED stage. **(D)** No significant correlation between baseline eye-blink rate and errors during the ED stage.

Baseline pupil size was significantly and negatively correlated with the number of errors in the ED stage, *r_s_* (42) = -0.35, *p* = 0.02, whereas it was not related to errors preceding the ED stage, *r_s_* (42) = -0.02, *p* = 0.86 (see **Figures 3A,B**).

In contrast to pupil size, the EBR was not associated with errors during the ED stage, *r_s_* (42) = -0.02, *p* = 0.92, but was significantly and positively correlated with errors preceding the ED stage, *r_s_* (42) = 0.46, *p* = 0.002 (see also **Figures 3C,D**). This correlation might have been confounded by the fact that EBR was steadily increasing throughout the task, and thus EBR was also computed by involving data from the beginning of the task, between the SDR and the CD2 stage. This alternative computation of EBR did not influence our results (correlation with errors before the ED stage: *r_s_* (42) = 0.37, *p* = 0.01; correlation with errors during the ED stage: *r_s_* (42) = -0.07, *p* = 0.66).

### Analysis of Rule Shifts – Data During the Fixation Cross Period

**FIGURE 4:**
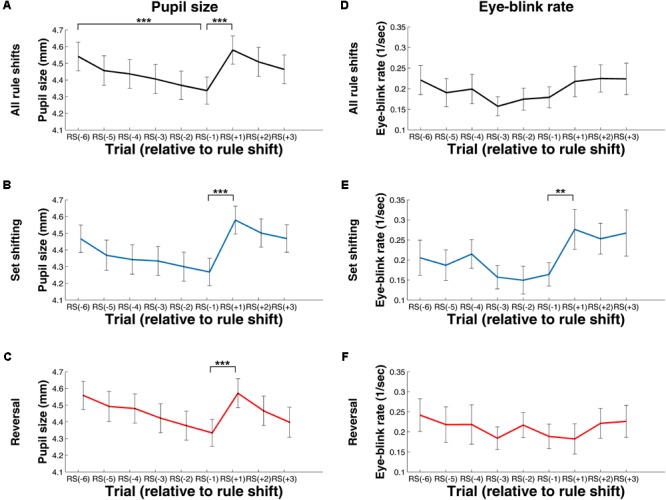
Change in Pupil Size **(A–C)** and Eye-Blink Rate **(D–F)** during Rule Shifts – Measures Computed for the Fixation Cross Period. **(A,C)** Values computed using data from all rule shifts. **(B,E)** Values computed using data from rule shifts involving attention set shifting. **(C,F)** Values computed using data from rule shifts involving reversal. RS[–1], RS[–2], (…), RS[–6], the first, second, (…), and the sixth trial, respectively, preceding the rule shift; RS[+1], RS[+2], RS[+3], the first, second, or third trial, respectively, following the rule shift. ^∗∗^*p* < 0.01; ^∗∗∗^*p* < 0.001.

**Figure 4** shows pupil size and EBR values before and during rule shifts. These values were measured during the fixation cross period, and thus are not conflated by effects related to visual features of the presented stimuli.

We found a significant decrease in pupil size during the exploitative phase (see **Figure 4A**). The main effect of trial was significant, *F*(2.91, 119.17) = 39.97, *p* < 0.001 (after Greenhouse–Geisser correction, epsilon = 0.58), η_p_^2^= 0.49, as was the linear trend, *F*(1, 41) = 75.04, *p* < .001, η_p_^2^= .65. There was a significant increase in pupil size between RS[–1] and RS[+1], *t*(41) = 10.99, *p* < .001, *d* = 1.69 (see **Figure 4A**). This difference was also present when focusing on rule shifts with attentional set shifting, *t*(41) = 8.59, *p* < .001, *d* = 1.32, and also when examining rule shifts with reversals, *t*(41) = 9.49, *p* < .001, *d* = 1.47 (see **Figures 4B,C**).

Regarding EBR, we did not find any difference during the trials of the exploitative phase, *χ^2^*(5) = 7.98, *p* = 0.16, and there was also no significant change between RS[–1] and RS[+1], *Z* = 1.52, *p* = 0.13, *r* = 0.16 (see **Figure 4D**). When restricting our data to rule shifts involving attentional set shifting, there was a significant increase in EBR after rule shift, *Z* = 2.75, *p* = 0.006, *r* = 0.30 (see **Figure 4E**). This was, however, not the case for the trials involving reversal, *Z* = 0.61, *p* = 0.55, *r* = 0.06 (see **Figure 4F**).

### Analysis of Rule Shifts – Data During the Stimulus Presentation and Feedback Period

**FIGURE 5:**
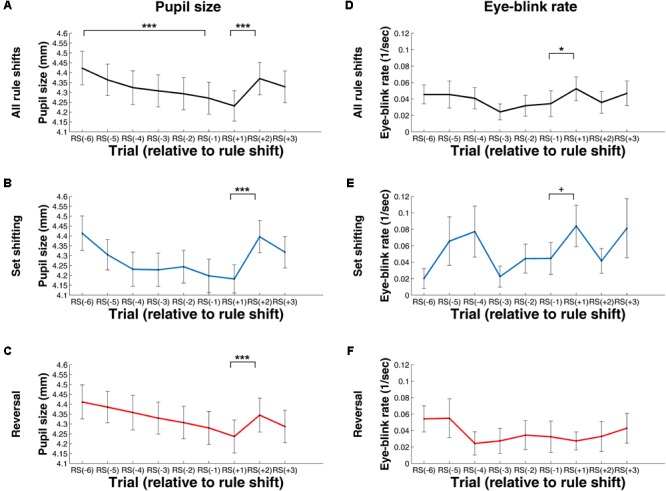
Change in Pupil Size **(A–C)** and Eye-Blink Rate **(D–F)** during Rule Shifts – Measures Computed for the Stimulus Presentation Period. **(A,C)** Values computed using data from all rule shifts. **(B,E)** Values computed using data from rule shifts involving attention set shifting. **(C,F)** Values computed using data from rule shifts involving reversal. RS[–1], RS[–2], (…), RS[–6]: the first, second, (…), and the sixth trial, respectively, preceding the rule shift; RS[+1], RS[+2], RS[+3]: the first, second, or third trial, respectively, following the rule shift. +*p* < 0.10; ^∗^*p* < 0.05; ^∗∗∗^*p* < 0.001.

**FIGURE 6:**
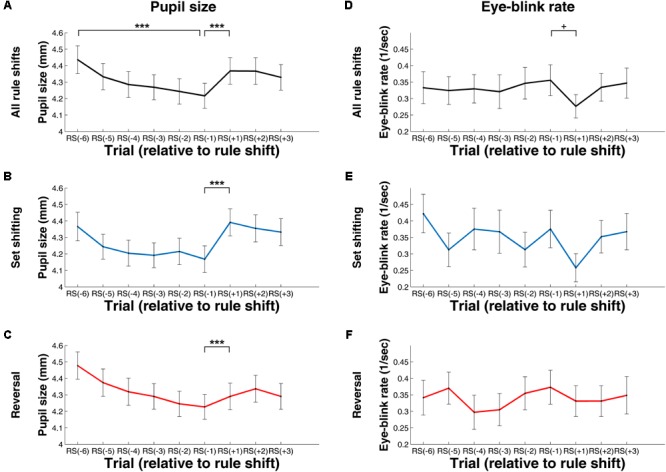
Change in Pupil Size **(A–C)** and Eye-Blink Rate **(D–F)** during Rule Shifts – Measures Computed for the Feedback Period. **(A,C)** Values computed using data from all rule shifts. **(B,E)** Values computed using data from rule shifts involving attention set shifting. **(C,F)** Values computed using data from rule shifts involving reversal. RS[–1], RS[–2], (…), RS[–6], the first, second, (…), and the sixth trial, respectively, preceding the rule shift; RS[+1], RS[+2], RS[+3], the first, second, or third trial, respectively, following the rule shift. +*p* < 0.10; ^∗∗∗^*p* < 0.001.

**Figures 5, 6** show pupil size and EBR values, computed for the stimulus presentation and the feedback period. Importantly, during these phases, both neurotransmitter levels and visual features might have determined pupil size and EBR.

#### Stimulus Presentation Period

A repeated measures ANOVA with trial as within-subject factor (RS[–6] to RS[–1]) showed a significant main effect of trial, *F*(3.26, 133.58) = 20.35, *p* < 0.001 (after Greenhouse–Geisser correction, epsilon = 0.65), η_p_^2^= 0.33. The linear contrast was also significant, *F*(1, 41) = 39.03, *p* < .001, η_p_^2^= 0.49 (see **Figure 5A**). Contrary to our hypothesis, there was a significant decrease in pupil size between RS[–1] and RS[+1], *t*(30) = 2.31, *p* = 0.03, *d* = 0.35. Interestingly, however, the predicted increase in pupil size occurred during the next trial: the pupil size during the RS[+2] trial was significantly higher, than the pupil size during the RS[+1] trial, *t*(41) = 7.97, *p* < .001, *d* = 1.23 (see **Figure 5A**). This lag might be explained by the fact that RS[+1] is the first time point where the participant can notice a rule shift, and thus changes in pupil size induced by this rule shift can be observed at the next trial. The increase between RS[+1] and RS[+2] was significant both for trials with stimulus-attentional set shifting, *t*(41) = 7.16, *p* < 0.001, *d* = 1.21, and for trials with reversals, *t*(41) = 3.81, *p* < 0.001, *d* = 0.59 (see **Figures 5B,C**).

The Friedman ANOVA tests suggest that EBR did not significantly change during the exploitative phase, *χ^2^*(5) = 9.01, *p* = 0.11, but there was a significant increase in EBR between RS[–1] and RS[+1], as indicated by the Wilcoxon signed rank test, *Z* = 2.29, *p* = 0.02, *r* = 0.24 (see **Figure 5D**). This increase disappeared when restricting our analysis either to rule shifts with attentional set shifting, *Z* = 1.78, *p* = 0.07, *r* = 0.19 (see **Figure 5E**), or to rule shifts involving reversals, *Z* = 0.17, *p* = 0.87, *r* = 0.01 (see **Figure 5F**).

#### Feedback Period

There was a significant decrease in pupil size during the exploitative phase, as evidenced by the significant main effect of trial, *F*(2.25, 92.27) = 49.08, *p* < 0.001 (after Greenhouse–Geisser correction, epsilon = 0.45), η_p_^2^= 0.55, and by a significant linear trend, *F*(1, 41) = 70.88, *p* < 0.001, η_p_^2^ = 0.634 (see **Figure 6A**). At the beginning of the explorative phase, the size of the pupil increased, as evidenced by a significant difference between RS[–1] and RS[+1], *t*(41) = 7.72, *p* < 0.001, *d* = 1.19 (see **Figure 6A**). This increase was evident for rule shifts with attentional set shifting, *t*(41) = 6.06, *p* < 0.001, *d* = 0.92 (see **Figure 6B**), and also for rule shifts with reversals, *t*(41) = 3.89, *p* < 0.001, *d* = 0.60 (see **Figure 6C**).

There was no significant difference in EBR during the exploitative phase, *χ^2^*(5) = 5.12, *p* = 0.39 (see **Figure 6D**). There was a non-significant tendency for a decrease in EBR between RS[–1] to RS[+1], *Z* = 1.69, *p* = 0.09, *r* = 0.18 (see **Figure 6D**). This decrease was not significant for either rule shifts with attentional set shifting, *Z* = 1.47, *p* = 0.14, *r* = .16 (see **Figure 6E**), and also not for rule shifts with reversals, *Z* = 0.97, *p* = 0.33, *r* = 0.10 (see **Figure 6F**).

### Analysis of the Effect of Potential Confounders

Measures of last night’s sleep, task effort, and drowsiness were not correlated with baseline measures of pupil size and EBR. Excluding participants with minor flu or concurrent medication only altered the results of significance testing for some of the results reported for EBR changes during rule shifts. Our most relevant result here was that EBR during the fixation cross period increased significantly for rule shifts requiring attentional set shifting. This difference remained significant, suggesting that the exclusion of participants merely decreased statistical power, and neither medication nor health condition confounded the results.

## Discussion

In this study, we used easily accessible physiological measures to investigate how indirect measures of NA and DA transmission are related to performance in a task assessing one specific aspect of EF, attentional set shifting. We found that individual differences in baseline levels of pupil size and EBR were associated with different aspects of task performance. Additionally, we also showed that within-task changes in pupil size and EBR reflected the transition between the exploitative and the explorative phase of the task. Below we outline these results and their potential implications in more detail.

First, we showed that baseline pupil size was related to performance in the ED stage. This result is a replication of the interesting finding of Tsukahara et al. (2016), who showed that individual differences in baseline pupil size were associated with performance in complex working memory tasks. Importantly, we found a specific association: baseline pupil size was only related to errors during the ED stage, but not to errors prior to this stage. This pattern of results suggests that baseline pupil size is specifically related to cognitive flexibility, and not to task effort or reinforcement learning. As proposed by Tsukahara et al. (2016), this correlation might be explained by the fact that baseline pupil size reflects the activity of the LC/NA system, which regulates the dynamics of different brain networks (Yellin et al., 2015; Shine et al., 2016), which in turn determine EF (Keller et al., 2015).

Second, we found a correlation between baseline EBR and task performance. We showed that individual differences in participants’ EBR were correlated with more erroneous choices preceding the ED stage. This correlation is in line with models suggesting that baseline EBR reflects dopaminergic regulation of the trade-off between maintaining versus updating working memory representations (Frank and O’Reilly, 2006; Cools and D’Esposito, 2011; Maia and Frank, 2011; Jongkees and Colzato, 2016). The IEDT requires the maintenance of the same attentional set in working memory during a line of subsequent choices. Low threshold for updating these representations, signaled by high EBR, leads to incorrect choices and this is reflected in the correlation between EBR and erroneous choices.

Third, we replicated our previous findings presented in Pajkossy et al. (2017): tonic pupil size, as measured during the fixation cross period, decreased steadily during the exploitative phase and increased when the explorative phase began. Moreover, we extended this finding by showing that this pattern was also present when we assessed pupil size during the stimulus presentation and the feedback period. Thus, change in tonic pupil size during rule shifts was a robust indicator of exploration and exploitation, and this change was not affected by other factors (e.g., changes in luminance during stimulus presentation or feedback processing). Furthermore, tonic increase in pupil size was present during both reversal and attentional set shifting. This pattern of results is in line with the suggestion of Yu and Dayan (2005), who proposed that tonic NA signals unexpected uncertainty. The increase in tonic pupil size following rule shifts signals uncertainty because the established stimulus–response contingencies are no longer valid. Once this uncertainty vanishes, during the exploitative phase, tonic NA (and so pupil size) starts to decrease.

Fourth, we observed task-dependent tonic changes in blinking behavior. By measuring EBR during the fixation cross period, we detected an increase in EBR after rule shifts. Interestingly, however, this change was only present during rule shifts involving attentional set shifting, but not after rule shifts involving reversal. An important difference between reversal and attention set shifting is the differential requirement of explorative processes. On the one hand, reversal stages do not require exploration, because after the first reversal, participants learn the logic of the task: negative feedback without change in stimulus display requires the reversal of response tendencies. On the other hand, in attentional set shifting stages, stimulus–response mappings have to be newly established, and this requires exploring which stimulus feature is associated with reward. Therefore, the selective association between change in EBR and attention set shifting is in line with suggestions, which link tonic DA level to explorative processes (Frank et al., 2009; Beeler et al., 2010; Humphries et al., 2012).

To sum up, we have demonstrated that pupil size and EBR are related to task performance in a way that is predicted by theories of NA and DA neurotransmitter system. Interestingly, baseline and tonic measures of these physiological variables were related to different aspects of task performance. Baseline pupil size was specifically related to the ED stage, whereas changes in tonic pupil size were observed after each rule shift. Similarly, baseline EBR was associated with performance preceding the ED stage, whereas tonic change of EBR was associated with performance during the ED stage. These discrepancies suggest that these physiological measures assess different aspects of NA and DA neurotransmission. Both neurotransmitter systems exert their effect on multiple time scales and in multiple brain sites involving different receptor types (see e.g., Aston-Jones and Cohen, 2005; Björklund and Dunnett, 2007). This complexity might be reflected in different aspects of these indirect physiological measures. The investigation of this issue poses an interesting challenge for future studies.

### Methodological Considerations

Our results also highlight that pupil size and eye-blinks are influenced by several factors; thus care should be taken to control for these confounders. For example, visual/structural features of the task can influence the pattern of results, as demonstrated by the EBR data from the stimulus presentation and the feedback period. The first feedback trial of a stage requires increased visual processing of the stimuli, and this can explain the decrease in EBR (see Drew, 1951; Stern and Skelly, 1984). Similarly, the increase of EBR during stimulus presentation after rule shifts was confounded by the fact that the time of the stimulus presentation was not fixed (as it was terminated by the participant’s response). Such visual/structural confounds cannot explain changes in EBR (and pupil size) during the fixation cross period; thus they can be interpreted as reflecting changes in DA (and NA) transmission.

The effect of stimulus luminance and room illumination on pupil size is also a potential confounding factor, which has to be carefully investigated before interpreting pupil size results. Although room illumination was held constant, stimulus luminance changed during the task, as the luminance of the figures and the background was different. To take this potential confounder into account, we only compared pupil size measures during the same trial period, where stimulus luminance conditions were comparable. During the fixation cross period, screen display was always the same; thus luminance could not cause the pattern of our results. During stimulus presentation and feedback phase, the stimulus display varied as different figures were presented. Note however, that the surface of each figure was the same, and so the net luminance of the screen remained constant. Because of this, it is unlikely that luminance differences would have influenced our results.

Another type of confound is related to participant characteristics and behavior. Pupil size and EBR are physiological variables, which are influenced by health condition, medication, arousal levels, nicotine/caffeine consumption, sleep quality, and sleep hours (Holmqvist et al., 2011). As it was described in detail in the result section, we verified that our results are not confounded by these variables.

Before interpreting pupil size and EBR as indirect measures of NA and DA transmission, we also carefully considered two specific measurement issues. First, it is important to note that eye-blink detection was performed indirectly, by analyzing gaze parameters measured by an eye-tracker. Therefore, measurement noise and other eye-tracking artifacts might have distorted eye-blink detection. To prevent this, we used a strict criterion to exclude participants with inappropriate data quality. Furthermore, distorted eye-blink detection would have caused a general over- or underreporting of EBR (due to false or missed detection of eye-blinks). Such bias is not likely to cause the specific pattern of our results (e.g., selective increase in EBR only after attentional set shifting, detected only during the fixation cross period). The second issue is related to the fact that pupil size and EBR are not completely independent measures. On the one hand, it has been shown that eye-blinks are followed by a sequence of short dilation and constriction of the pupil (Knapen et al., 2016). On the other hand, pupil size influences the quality of those gaze data sets (Choe et al., 2016), which are used to detect eye-blinks. These interdependencies offer the possibility that changes in pupil size and EBR do not reflect the distinct influence of the NA and the DA neurotransmitter systems; instead the change in one measure is caused by the change in the other. The specific pattern of our results, however, contradicts this interpretation. Pupil size and EBR do change in a similar way during the fixation cross period, but not during the stimulus-presentation and the feedback periods. If pupil size and EBR values would have been influenced by some common measurement artifacts, this would have affected them similarly in all periods.

Finally, it is important to highlight a limitation of our results, with respect to the baseline EBR measure we used. We measured baseline EBR not during a passive viewing condition, but during the task. Admittedly, several factors might influence EBR values during task execution (Irwin and Thomas, 2010; Jongkees and Colzato, 2016), and these factors might have confounded our EBR measure. Nevertheless, our results are in line with previous studies using EBR under free viewing conditions, which found that EBR is linked to the trade-off between maintaining and updating cortical representations (e.g., Dreisbach et al., 2005; Zhang et al., 2015; for a review see Jongkees and Colzato, 2016). This might raise the possibility that EBR during task execution can be also used as an indirect measure of DA transmission – this assumption should be tested in future studies, for example by assessing EBR during both a free viewing and a task execution condition.

## Conclusion

In summary, our results show that easily accessible physiological indexes can be used to assess how NA and DA transmission is associated with attentional set shifting. We have demonstrated that individual differences in pupil size and EBR are correlated with individual differences in task performance. Moreover, we also showed that within-task changes of pupil size and EBR might reveal how changes in NA and DA transmission accompanies exploitative and explorative aspects of attentional set shifting. Importantly, the pattern of our results showed a specific relationship between different task features (e.g., stages involving reversal vs. attentional set shifting) and different aspects of the physiological variables (e.g., baseline level vs. within-task changes). These results suggest that the NA and DA neurotransmitter systems are involved in attentional set shifting by regulating the balance between different and antagonistic aspects of information processing required during the task (exploration vs. exploitation, stability vs. flexibility). Our results suggest that by measuring pupil size and EBR, we can shed light on how variations in EF are related to variations in neurotransmitter levels, and thus this method might be a promising tool in exploring the sources of variability in EF.

## Ethics Statement

The protocol was approved and the study was carried out in accordance with the recommendations of the United Ethical Review Committee for Research in Psychology, Hungary. All subjects gave written informed consent in accordance with the Declaration of Helsinki.

## Author Contributions

PP, ÁS, GD, and MR participated in planning and designing the experiment. PP collected and analyzed the data and wrote the first version of the manuscript, which was then discussed with ÁS, MR, and GD before preparing the final version.

## Conflict of Interest Statement

The authors declare that the research was conducted in the absence of any commercial or financial relationships that could be construed as a potential conflict of interest.

## Footnotes

1 We define baseline measure as an index which has a relative stability over time, whereas tonic changes are conceptualized as changes evolving over a relatively slow time scale, over seconds (in contrast to phasic changes, which evolve in the seconds-milliseconds time range). We follow hereby the terminology of the adaptive gain theory which differentiates between phasic and tonic change in LC firing patterns. Note, however, that different terminology can also be used: the measure termed baseline EBR by us is referred to as tonic EBR in Jongkees and Colzato (2016), whereas changes in EBR during a task is termed tonic change by us, but phasic change in Jongkees and Colzato.
